# Endobronchial Valve Insertion for the Management of Persistent Air Leak Following Pneumothorax

**DOI:** 10.7759/cureus.74071

**Published:** 2024-11-20

**Authors:** Seemab Paul, Katherine Reid, Vasileios Lostarakos

**Affiliations:** 1 Respiratory Medicine, South Tyneside and Sunderland NHS Foundation Trust, Sunderland, GBR; 2 Internal Medicine, South Tyneside and Sunderland NHS Foundation Trust, Sunderland, GBR

**Keywords:** chest drain, endobronchial valves, fistula, persistent air leak, pneumothorax

## Abstract

Persistent air leak following a pneumothorax refers to air leakage lasting 5-7 days after the initial event. Different strategies have been used with varying degrees of success including surgical or chemical pleurodesis. Endobronchial valve (EBV) insertion is a technique where the insertion of a one-way valve obstructs the flow of air through the leak and helps in pneumothorax resolution especially if surgery is contraindicated.

We present the case of a gentleman in his 60s who was admitted with a right-sided pneumothorax on the background of grade 4 glioblastoma. A 12-French chest drain was inserted for the management of his pneumothorax. A CT scan was requested due to failure of resolution of pneumothorax at five days post chest drain insertion, evidenced by ongoing bubbling in the underwater seal. CT revealed moderate right-sided pneumothorax and a possible bronchopleural fistula arising from the right upper lobe posterior segment bronchiole. He was unfit for surgery. A second chest drain was inserted but the air leak did not settle.

It was then decided to insert EBV using bronchoscopy under sedation and Zephyr valves were used for this procedure. Balloon blockage of the right upper lobe led to the disappearance of air leak in the underwater seal on -5 kPa suction whereas blocking individual branches did not lead to the termination of air leak, therefore, it was decided to insert valves on all the segments of the right upper lobe. Four days after the EBV insertion his air leak resolved and his chest X-ray (CXR) showed a resolution of pneumothorax, so his chest drain was removed. He was subsequently discharged home.

EBV insertion can facilitate pneumothorax resolution and discharge in carefully selected patients and should be used with a multi-disciplinary approach. In our case, this approach helped to facilitate the transfer of a terminally ill patient to his preferred place of comfort.

## Introduction

An air leak is defined as the flow of air into the pleural space, generally through a fistulous tract either in the periphery of the lung (alveolar-pleural fistula) or in the more central airways (bronchopleural fistula). Definitions of persistent air leak following pneumothorax differ. A commonly used definition is that of an air leak lasting 5-7 days, whilst others define it as lasting more than 48 hours [1]. While usually caused post surgery, e.g. lobectomy, these can also be caused secondary to trauma, lung biopsy, pulmonary infections and emphysema. Different strategies have been used with varying degrees of success which include surgical or chemical pleurodesis.

One-way endobronchial valve (EBV) insertion is a technique used for persistent air leak which aims to find the location of air leakage and insert a one-way valve which obstructs the flow of air through the leak and provides time for the pulmonary defect to heal [2]. It is particularly useful in patients where there is a contraindication to surgical procedures or general anaesthesia. A case series performed by Travaline et al. where 40 patients with persistent air leak were treated with EBV insertion reported a complete resolution of air leak in 19 patients (47.5%), reduction of air leakage in 18 patients (45%) and no change in two patients (5%) [3]. In a recent meta-analysis of 28 observational studies performed by Damaraju et al., an overall success rate of 82% was seen for bronchial valve insertion (intrabronchial and endobronchial) for persistent air leak. Complications were seen in 9.1% of all cases with granulation tissue formation seen most followed by valve displacement and hypoxaemia [4]. Both the British Thoracic Society (BTS) and National Institute for Healthcare Excellence (NICE) have issued guidance on the potential use of this treatment in carefully selected patients with persistent air leak. The guidance states that although there is limited evidence for the efficacy of EBV insertion in persistent air leak, it can be used in certain cases following a multidisciplinary team discussion, particularly if alternatives are not available [5,6].

## Case presentation

**Figure 1:**
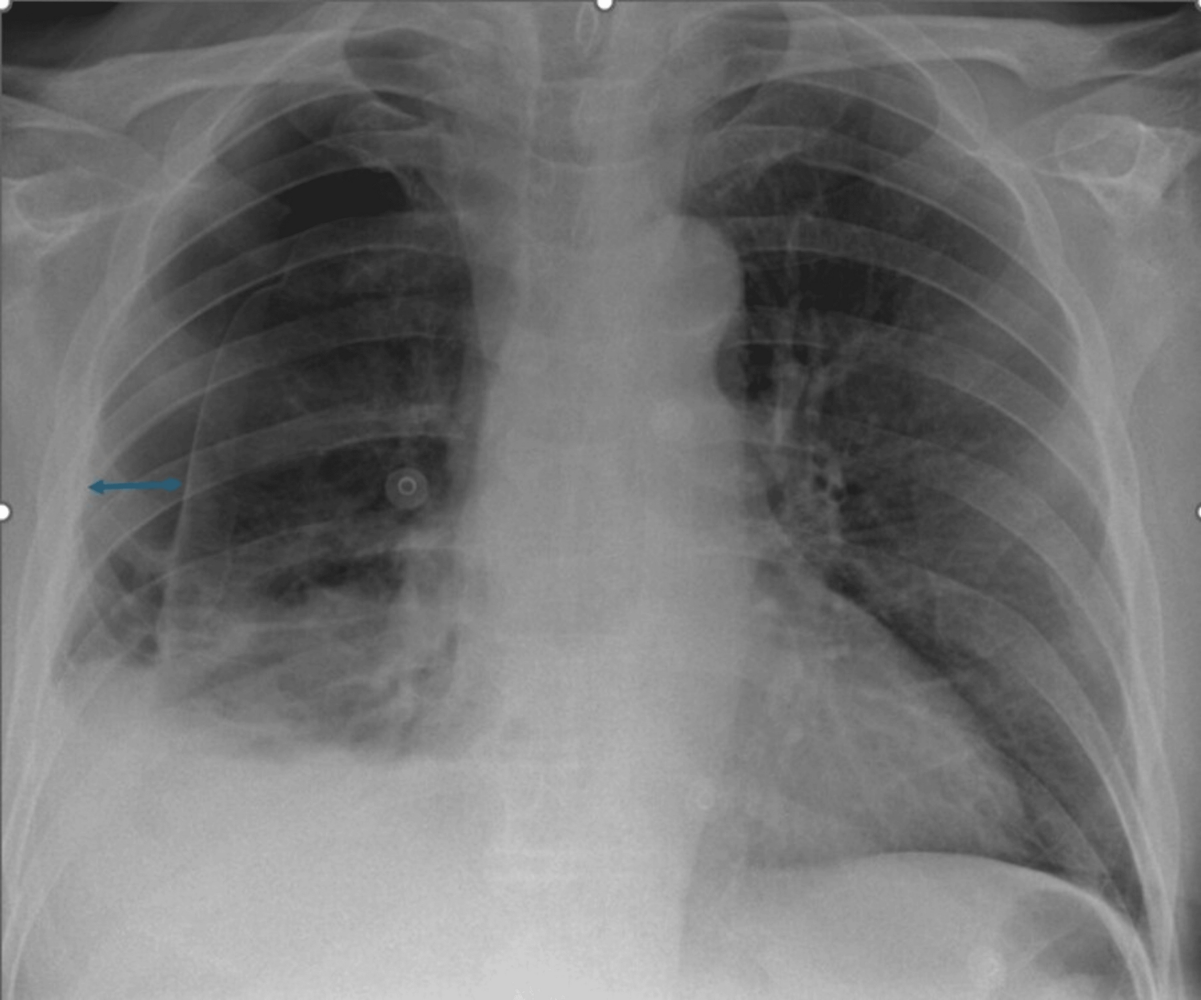
Chest X-ray showing right-sided pneumothorax at presentation

This is a case of a gentleman in his 60s who presented with a right-sided pneumothorax on a background of Grade 4 glioblastoma for which he started receiving chemotherapy with temozolomide but his last cycle was interrupted due to his hospital admission. He presented to the Emergency Department (ED) with a few weeks history of progressive dyspnoea, acutely worsening two days prior to presentation, in the absence of chest pain or cough. He had no diagnosed chronic obstructive pulmonary disease (COPD). He required oxygen at 2 litres/minute via nasal cannula. His chest X-ray (CXR) on admission revealed a right-sided (R) pneumothorax measuring 2.9 cm at the hilum (Figure 1).

Because of his symptoms, a 12 French (Fr) chest drain was inserted via the triangle of safety and he was commenced on 15 litres/minute high flow oxygen via a non-breather mask (NBM) to supplement the resolution of the pneumothorax. He was also commenced on Amoxicillin for potential concurrent infection.

**Figure 2:**
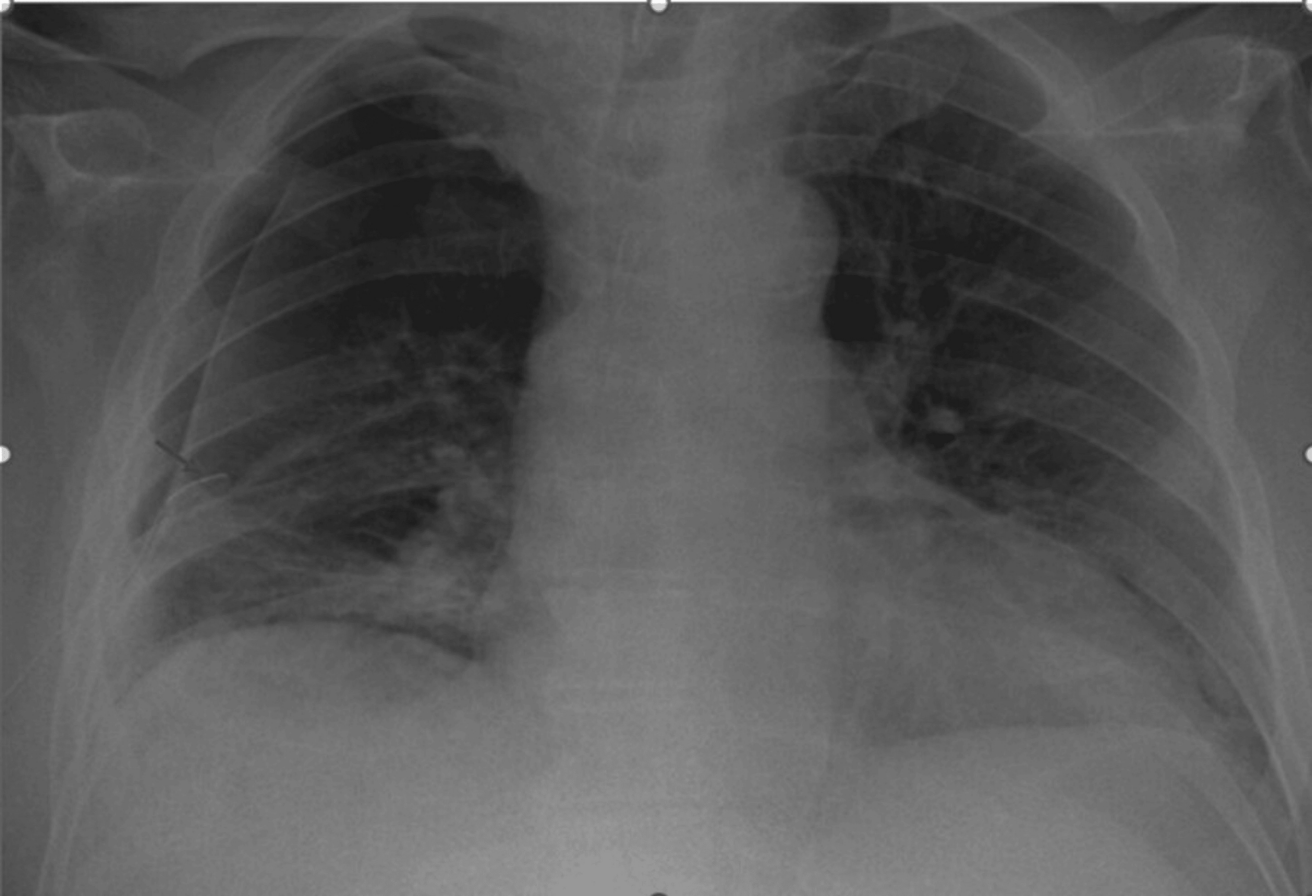
CXR taken five days post chest drain insertion showing persistent pneumothorax and chest drain in-situ (marked by arrow)

**Figure 3:**
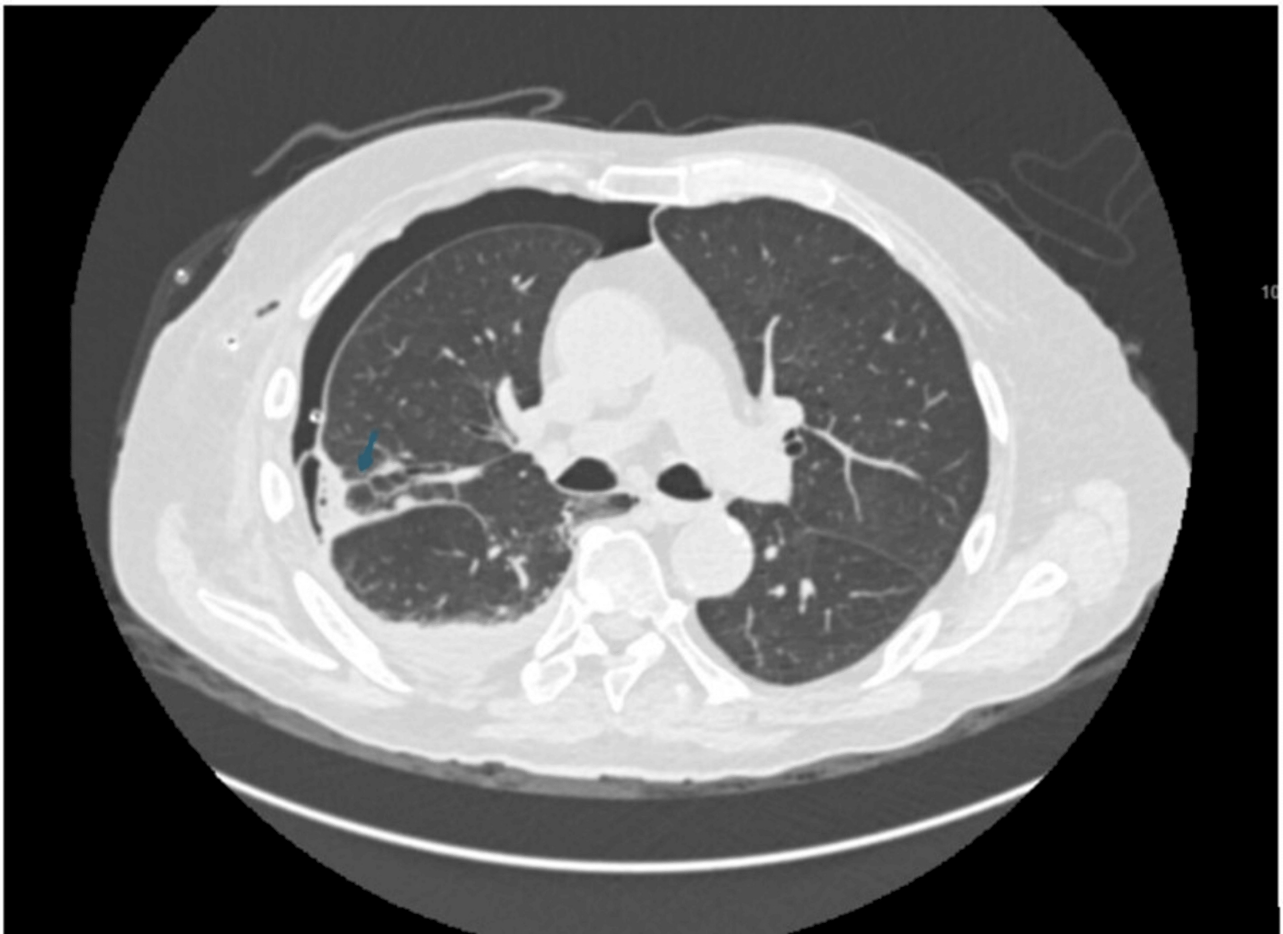
CT scan done five days post chest drain insertion showing possible bronchopleural fistula (BPF) arising from right upper lobe posterior segment bronchiole (marked by arrow)

Five days post chest drain insertion there was ongoing bubbling in the under-water seal and a repeat CXR showed the failure of resolution of his pneumothorax, despite suction at -2.5 kPa (Figure 2). A CT thorax was subsequently performed which showed minor emphysema and a moderate-sized right pneumothorax despite an adequately sited pleural catheter and a possible bronchopleural fistula arising from the right upper posterior segment bronchiole (Figure 3). The on-call cardiothoracic surgery team was contacted who advised that the fistula is non-correctable operatively; moreover, his malignant glioblastoma makes him a high-risk candidate for general anaesthesia (GA), so this precludes surgical options.

**Figure 4:**
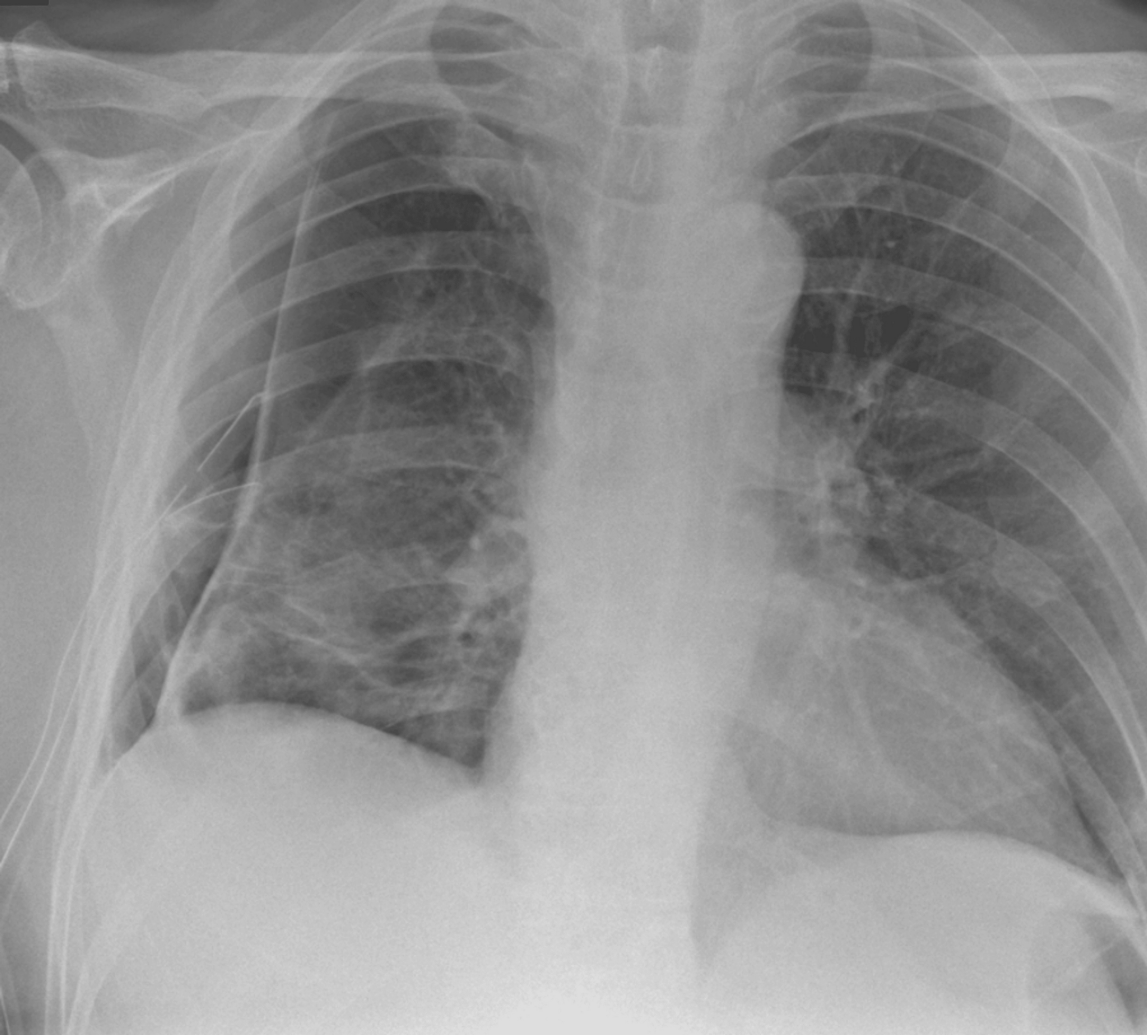
CXR showing failure of pneumothorax to resolve despite insertion of two chest drains

Seven days after initial presentation a second 18 Fr chest drain was inserted into the right mid-axillary line under ultrasound guidance, in addition to the first drain. Despite this, there was persistent air leak evidenced by persistent bubbling in the under-water seal and little improvement on repeat CXR after two weeks of his initial presentation (Figure 4).

In light of the above, following a multi-disciplinary approach, a decision was made, along with the patient and his family, to proceed to endobronchial valve (EBV) insertion using bronchoscopy under sedation with Zephyr valves. One of the chest drains had dislodged on mobilisation and so there was only one chest drain remaining at the time of EBV insertion. At the time of bronchoscopy, an examination of the right upper lobe revealed four segmental branches which is atypical. The balloon blockage technique was used to determine the source of air leak. Each of the four segments was blocked individually by inflating a balloon placed at the end of the bronchoscope and an air leak was observed in the underwater seal connected to his chest drain, revealing that blocking one of these segments will not lead to the resolution of the air leak. However, balloon blockage of the right upper lobe bronchus led to the termination of air leak. Therefore, all four segments were blocked using four endobronchial valves. This was carried out without complication.

**Figure 5:**
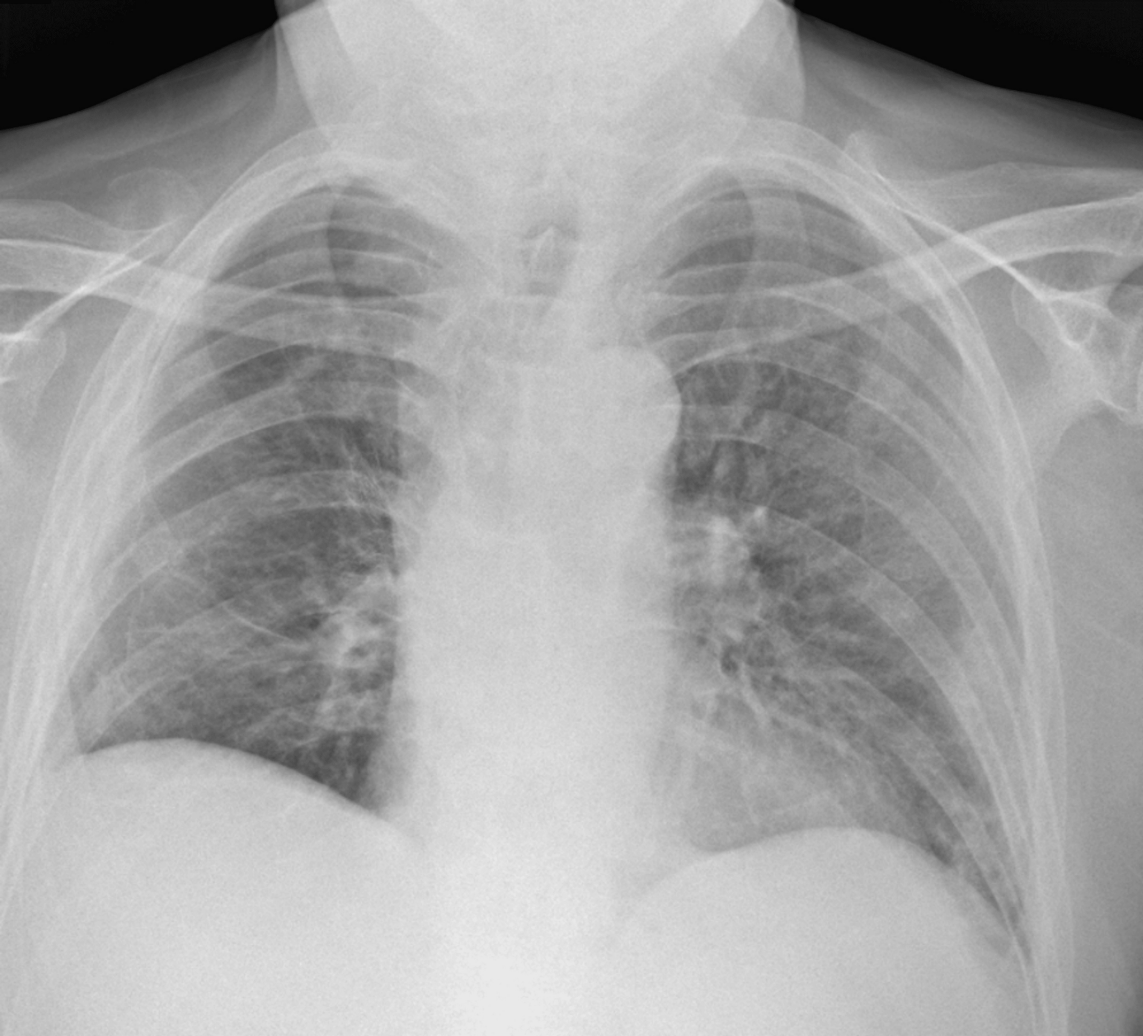
CXR showing resolution of the right-sided pneumothorax after EBV insertion EBV: Endobronchial valve

Four days later, his air leak had resolved and repeat CXR showed a resolution of pneumothorax (Figure 5). The chest drain was removed at this point. He was able to be discharged home eight days post EBV insertion with oncology and neurology input regarding his glioblastoma and secondary seizures.

**Figure 6:**
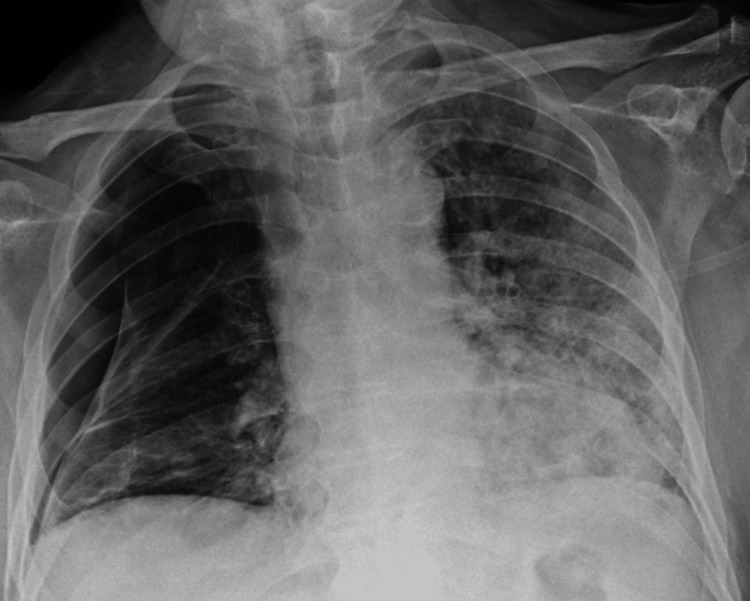
CXR showing recurrence of right pneumothorax three weeks post EBV insertion along with increasing shadowing in the left lung EBV: Endobronchial valve

Unfortunately, he was re-admitted approximately two weeks later (three weeks post EBV insertion) with progressive dyspnoea and general decline. Repeat CXR revealed a recurrence of his right pneumothorax (Figure 6). Given his underlying co-morbidities and decline it was decided that he was too frail for further chest drain/procedures and management was moved towards palliative care. This aligned with his previous wishes and that of his family as his preferred place of care was home. He sadly passed away soon thereafter.

## Discussion

This case highlights the benefit and importance of endobronchial procedures in patients with persistent air leak who are not surgical candidates. In the case discussed above, we were able to control the air leak and discharge the patient to the comfort of his home. Chest drain in situ is a source of discomfort for patients and we were able to remove it prior to his discharge.

EBVs are devices that act as one-way valves sealing or blocking the flow of air to the desired lung area during inspiration but allowing air to flow out during expiration. Two types of EBVs have been approved by the Food and Drug Authority (FDA) since 2018, namely Zephyr and Spiration valves [7]. While initially approved for COPD as a management option for emphysema, they can also be used to control persistent air leak in pneumothorax where surgical options are not suitable [8].

The risks with EBVs include valve migration and even expectoration, recurrent chest infections, haemoptysis, and granulation tissue formation over the valves. These may necessitate the removal of EBVs and serve as a limiting factor towards their use [5].

In our case, the patient was not a candidate for surgical procedure given his frailty and co-morbidities. Persistent air leak and having two chest drains were limiting his mobility, causing discomfort and delaying discharge. An ambulatory valve was considered but given the size of the pneumothorax, it was deemed inappropriate. EBV insertion was able to control the air leak which facilitated chest drain removal and discharge according to the patient's wishes and he was able to spend time in the comfort of his home.

His recurrent pneumothorax could be explained as being related to his underlying emphysema but the precise cause is difficult to ascertain in this case. Cases have been reported where patients had recurrence of air leak after initial resolution of temporary reduction [9]. However, a pooled success rate defined as complete resolution of air leak after EBV insertion and requiring no further procedure of 72% was observed by a meta-analysis analyzing 28 observational studies [4]. Overall it is less invasive, safer and better tolerated than surgical options.

## Conclusions

EBV insertion was first used for bronchoscopic lung volume reduction and its use is now expanding to treat persistent air leaks following multiple aetiologies such as first presentation of pneumothorax, recurrent, iatrogenic, traumatic and post-surgical pneumothorax. It is effective in managing persistent air leak when surgical options are less viable and can facilitate in chest drain removal and discharge of patients. This is a low-risk procedure done bronchoscopically under sedation and should be offered to suitable candidates after a multi-disciplinary team discussion.

This case report illustrates the utility of EBV to control persistent air leak for a patient where a surgical option was not available. More prospective studies with a standardized protocol are needed to determine the effectiveness of this procedure.

## References

[1] Lazarus DR, Casal RF (2017). Persistent air leaks: a review with an emphasis on bronchoscopic management. J Thorac Dis.

[2] Snell GI, Holsworth L, Fowler S, Eriksson L, Reed A, Daniels FJ, Williams TJ (2005). Occlusion of a broncho-cutaneous fistula with endobronchial one-way valves. Ann Thorac Surg.

[3] Travaline JM, McKenna RJ Jr, De Giacomo T, Venuta F, Hazelrigg SR, Boomer M, Criner GJ (2009). Treatment of persistent pulmonary air leaks using endobronchial valves. Chest.

[4] Damaraju V, Sehgal IS, Muthu V, Prasad KT, Dhooria S, Aggarwal AN, Agarwal R (2024). Bronchial valves for persistent air leak: a systematic review and meta-analysis. J Bronchology Interv Pulmonol.

[5] (2024). NICE. Insertion of endobronchial valves for persistent air leaks: Guidance [Internet]. https://www.nice.org.uk/guidance/ipg448/chapter/1-Guidance.

[6] Roberts ME, Rahman NM, Maskell NA (2023). British Thoracic Society Guideline for pleural disease. Thorax.

[7] American Lung Association (2024). American Lung Association. Endobronchial valve (EBV) therapy. https://www.lung.org/lung-health-diseases/lung-procedures-and-tests/ebv-therapy.

[8] Bermea RS, Miller J, Wilson WW, Dugan K, Frye L, Murgu S, Hogarth DK (2019). One-way endobronchial valves as management for persistent air leaks: a preview of what's to come?. Am J Respir Crit Care Med.

[9] Yu WC, Yu EL, Kwok HC (2018). Endobronchial valve for treatment of persistent air leak complicating spontaneous pneumothorax. Hong Kong Med J.

